# A Phase I, multicenter, open-label trial to evaluate the safety of talimogene laherparepvec (T-VEC) injected into liver tumors

**DOI:** 10.1186/2051-1426-3-S2-P180

**Published:** 2015-11-04

**Authors:** J Randolph Hecht, Steven Raman, Daniel Y Sze, A Craig Lockhart, Rebecca A Moss, Kate Liu, Jeffrey Chou, Tony Reid

**Affiliations:** 1 David Geffen School of Medicine at UCLA, Los Angeles, CA, USA; 2 Stanford University School of Medicine, Stanford, CA, USA; 3 Washington University School of Medicine, St. Louis, MO, USA; 4 Rutgers Cancer Institute of New Jersey, New Brunswick, NJ, USA; 5 Amgen Inc., Thousand Oaks, CA, USA; 6 Moores Cancer Center, University of California, San Diego, La Jolla, CA, USA

## Introduction

T-VEC, an intralesionally-delivered oncolytic immunotherapy, is a herpes simplex virus-1 engineered to selectively replicate in tumors and stimulate an anti-tumor immune response through expression of GM-CSF. T-VEC has the ability to lyse various cancer cell types in vitro[1]. A Phase III study of T-VEC injected into skin, subcutaneous, or lymph node tumors versus subcutaneous GM-CSF in advanced melanoma demonstrated improved durable response rate for T-VEC, with regression of both injected and uninjected lesions[2]. To further explore if different types of cancers and locations might be treatable with T-VEC, this Phase I study evaluates whether primary and metastatic liver tumors may be safely and effectively injected with T-VEC.

## Methods

Approximately 100 patients will be enrolled. Primary objective: evaluate maximum tolerated dose (MTD) of intrahepatic injection of T-VEC by patient incidence of dose-limiting toxicities (DLTs). Key secondary objectives: overall safety, efficacy, and biodistribution of T-VEC. Key eligibility criteria: breast, colorectal, gastroesophageal, kidney, lung cancer or melanoma with liver metastases (non-HCC) or hepatocellular carcinoma (HCC); measurable liver tumors suitable for injection; ECOG performance status 0-1; life expectancy ≥5 months; ≥1 prior standard systemic anticancer therapy (non-HCC); Child-Pugh A-B7; no detectable hepatitis B/C viral load; not a candidate for surgery or locoregional therapy of liver tumors with curative intent or planned systemic anticancer therapy; tumor in < 1/3 of the liver; no macroscopic intravascular invasion. The study consists of two parts. Part 1: 3+3 dose escalation of 3 sequential dose cohorts each administering T-VEC in increasing concentrations (10^7^ or 10^8^ PFU/mL) and volumes (up to 4 or 8 mL). MTD for HCC is determined separately from non-HCC tumor types; HCC cohorts will not proceed until safety at respective dose levels are determined in non-HCC. Six T-VEC doses injected under ultrasound or computed tomography guidance q21 (±3) days are planned, with an investigator option to continue for up to 6 additional doses. The first dose of T-VEC in all dose cohorts is given at 10^6^ PFU/mL. Part 2: 7 expansion cohorts for each cancer type with 10 patients each administered the MTD of T-VEC determined from Part 1.
